# Dysphagia screening after acute stroke: a quality improvement project using criteria-based clinical audit

**DOI:** 10.1186/s12912-017-0222-6

**Published:** 2017-06-02

**Authors:** Jorun Sivertsen, Birgitte Graverholt, Birgitte Espehaug

**Affiliations:** 1 Helse-Bergen, Haukeland University Hospital, Sjukehusvegen 16, 5704 Voss, Norway; 2 Centre for Evidence-Based Practice, Western Norway University of Applied Sciences, Postbox 7030, 5020 Bergen, Norway

**Keywords:** Criteria-based clinical audit, Swallow screening, Stroke, Dysphagia, Deglutition disorder

## Abstract

**Background:**

Dysphagia is common after stroke and represents a major risk factor for developing aspiration pneumonia. Early detection can reduce the risk of pulmonary complications and death. Despite the fact that evidence-based guidelines recommend screening for swallowing deficit using a standardized screening tool, national audits has identified a gap between practice and this recommendation. The aim was to determine the level of adherence to an evidence-based recommendation on swallow assessment and to take actions to improve practice if necessary.

**Methods:**

We carried out a criteria-based clinical audit (CBCA) in a small stroke unit at a Norwegian hospital. Patients with hemorrhagic stroke, ischemic stroke and transient ischemic attack were included. A power calculation informed the number of included patients at baseline (*n* = 80) and at re-audit (*n* = 35). We compared the baseline result with the evidence-based criteria and gave feedback to management and staff. A brainstorming session, a root–cause analysis and implementation science were used to inform the quality improvement actions which consisted of workshops, use of local opinion leaders, manual paper reminders and feedback. We completed a re-audit after implementation. Percentages and median are reported with 95% confidence intervals (CI).

**Results:**

Among 88 cases at baseline, documentation of swallow screening was complete for 6% (95% CI 2–11). In the re-audit (*n* = 51) 61% (95% CI 45–74) had a complete screening.

**Conclusion:**

A CBCA involving management and staff, and using multiple tailored intervention targeting barriers, led to greater adherence with the recommendation for screening stroke patients for dysphagia.

## Background

Globally, an estimate of 15 million people suffer from stroke annually. Of these, more than six million people die and five million develop a lifelong disability [1, 2]. Swallowing difficulties or dysphagia, is a common co-morbidity after acute stroke and affects 37 to 78% of all stroke-patients [3]. The incidence of dysphagia is highest early in the course of disease, decreasing from 51% at day zero to 27% at day seven [4]. There is a three times higher risk of developing pneumonia for stroke patients with dysphagia compared to patients without dysphagia [3]. As many as 22–52% of patients with dysphagia aspirate [3], and the risk of developing pneumonia is 11times higher for these patients compared to those who do not aspirate [3]. In addition, aspiration pneumonia is associated with a three times increased mortality risk compared to stroke patients without pneumonia [5]. The overall odds of malnutrition also increases with dysphagia [6]. Another challenge is that half of the stroke patients with dysphagia are unaware of their swallowing problems, which place them at high risk of aspiration and its associated consequences and such a lack of awareness correlates with health problems [7].

Despite the fact that clinical guidelines based on systematic reviews are available and clearly recommend screening for dysphagia before giving anything orally [8–11] several national audits have demonstrated a deficit in clinical practice [12–14]. To increase the adherence rate and the identification of dysphagia, studies have found that implementing a dysphagia screening protocol using a multifaceted implementation strategy can be effective [3, 15–18]. Consequently, early identification of dysphagia should be of high priority, to reduce the risks of co-morbidity, malnutrition and mortality [6, 19].

The aim of the project was to determine the level of adherence to an evidence-based recommendation on swallow assessment, in a small stroke unit at a Norwegian hospital, and to take actions to improve practice if necessary. We hypothesized that there was a gap between practice and recommendation, and that conducting a criteria-based clinical audit (CBCA) would improve practice.

## Methods

**Fig. 1:**
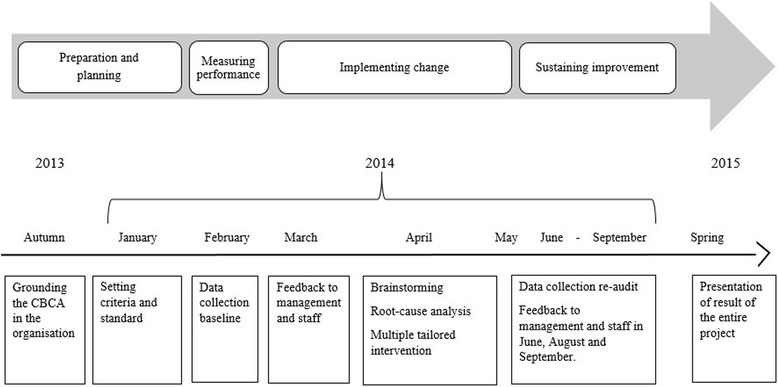
Course of the CBCA, from preparation and planning to sustaining improvement

Criteria-based clinical audit (CBCA) is a quality improvement cycle, where defined aspects of care is reviewed against evidence-based criteria, to evaluate the degree of adherence against these criteria and to implement necessary changes to practice [20] (Fig. 1). In CBCA, criteria reflects explicit recommendations from evidence-based guidelines and should not give room for interpretation. The stepwise model of Healthcare Quality Improvement Partnership HQIP [21] was used to conduct this quality improvement project. HQIP is an independent organisation promoting quality in healthcare.

### Setting, criteria and standard

Our setting was a small stroke unit at a Norwegian hospital. The stroke unit consists of four beds within a medical ward with 34 beds. On average, 90 stroke patients are submitted to the stroke unit annually. The unit is staffed with registered nurses who receive theoretical and practical training in the assessment of dysphagia. In 2011, The Norwegian Board of Health Supervision conducted a national audit of the treatment of elderly stroke patients. For our hospital, this resulted in remarks to deficits, and several measures were instigated to improve the quality of care. One measure was to introduce the swallow screening instrument, recommended in the Norwegian guideline on stroke [9]. We sat the criterion: All stroke patients (100%) with ICD-10 stroke diagnoses should be screened for swallow deficiency with a standardized swallow screening instrument recommended by The Norwegian Directorate of Health.

Process criteria do not measure the result of an activity, but the activity itself [22]. Our process criterion reflected nurses’ adherence to evidence best practice on swallow screening. This criterion was founded on recommendations in several national guidelines for stroke care [8–11], which was later confirmed in updated resources for clinical decision support [23]. The AGREE II instrument was used to assess the quality of the guidelines [24]. The overall quality was scored 5/7 and 6/7, respectively, for the Norwegian and Australian guidelines, implying they are both guidelines recommended for use.

### The swallow screening protocol

The swallow screening protocol recommended in the Norwegian guideline was based on consensus [9]. The screening instrument is a three-step test. The first step is to establish if the patient is eligible for the water swallow test, by assessing consciousness, the muscular control of trunk and head, as well as the patients’ ability to protect the airways. If this assessment is satisfactory, the second step is the water swallow test that can be performed with or without fluid thickener. In this step larynx elevation, and extended and multiple swallowing is observed while the patient swallows water from a teaspoon three times. Coughing, rattling voice and change in respiration is also observed at this step. If the patient pass the second step of the test, they continue to the third step where they are allowed to swallow 50 ml of water and a final assessment of swallow ability is made.

### Data and data-collecting tool

None of the guidelines we employed provided an audit tool, a data-collecting tool, to go with it. A search for one in the literature was unsuccessful, so we developed our own [25] ( Table 1). Our audit tool is based on the Australian and English audit on stroke performance and the swallow screening tool recommended in the Norwegian guideline [8, 9, 26].

**Table 1:**
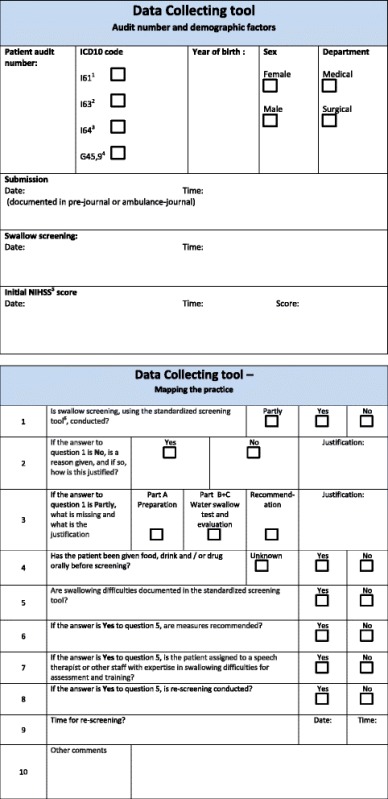
Data Collecting tool. Description of data: Data Collecting tool for demographic factors and mapping the practice

^1^ I61 – Non-traumatic intracerebral haemorrhage; ^2^ I63 – Cerebral infarction; ^3^ I64 – Stroke, not specified as haemorrhage or infarction; ^4^ G45,9 –Transient cerebral ischemic attack, unspecified (TIA); ^5^ NIHSS - National Institutes of Health Stroke Scale; ^6^ Recommended in the Norwegian guideline

The tool was designed to assess adherence to recommendations on swallow screening, and to collect the demographic variables time of admission, gender, age, type of stroke and stroke severity. Stroke severity was classified using the The National Institutes of Health Stroke Scale (NIHSS). The NIHSS scale range from zero to 42, where zero indicates no signs of the disease. The categories of severity were mild 0–8, moderate 9–16 and severe > 18 [27].

An expert panel of four nurses assessed the validity of the audit tool in a three-step procedure. The nurses were considered experienced in assessing stroke patients’ deglutition ability. The first step was a face validity check [25], where the nurses were asked if questions and formulations in the audit form seemed comprehensible and sensible. The panel had a positive subjective impression of the tool. Secondly, content validity was addressed using the content validity index (CVI). Each individual item of the tool (I-CVI), as well as the tool in its entirety (S-CVI) [28] was judged for relevance to the topic investigated. The expert panel used a 4-point scale, ranging from not relevant to highly relevant. The I-CVI was calculated as the proportion of experts that graded the item as “quite relevant” or “highly relevant” (scores 3 or 4), and the S-CVI as the proportion of items that all experts graded with scores of 3 or 4. Content validity was excellent [29], both on item-level and for the overall scale (CVI = 1.0). Third, we pilot tested the audit tool, where two expert nurses individually collected data in eight randomly selected electronic medical records (EMR). To select the EMRs we used the randomization program on the website https://www.random.org/. The purpose was twofold: We wanted to ensure that the audit tool was understandable and relevant, and secondly to check if we would find answers to the questions asked in the audit tool [25, 28, 30, 31]. The data collected by the two nurses were compared and assessed for inter-rater reliability [28]. The proportion of agreement for the audit tool was 93%. A written manual was developed to ensure a uniform use of the tool [31]. One person collected data from the patient’s EMR and the same person checked each record twice to identify errors [30]. For a patient to score “screened for dysphagia” a complete swallow screening had to be documented.

### Sample

To identify eligible stroke patients we searched the electronic patient system by the ICD-10 codes, I61-Non-traumatic intracerebral hemorrhage, I63-Cerebral infarction, I64-Stroke, not specified as haemorrhage or infarction and G45.9-Transient cerebral ischemic attack, unspecified (TIA). We excluded patients if they had a pre-existing swallowing problem prior to the acute incident. These patients were identified by having a feeding tube upon admission. A power calculation using IBM SPSS Sample Power 3 informed the number of included patients. As we anticipated a higher number of patients available for inclusion at baseline (retrospective audit data) than at re-audit, at least during a reasonable re-audit time frame, we allowed for unequal sample sizes (2:1). Thus, a total of 115 patients, 80 at baseline and 35 at re-audit were needed to detect an improvement from 10 to 35% as statistically significant (*p* < 0.05) with a power of 80%. We used a consecutive sampling plan. While baseline data (*n* = 90) was collected in retrospect comprising EMRs between December 2012 and January 2014, Re-audit data (*n* = 51) was collected prospectively from January 2014 to May 2014.

### Planning and assessing the implementation

Our implementation strategy was to develop a multifaceted intervention, largely based on implementation science to overcome local barriers towards the recommended practice on swallow assessment. To determine the barriers we organised a brainstorming session among the nurses in the unit [32]. To determine what caused the barriers and to identify the main subject, we performed a root-cause analysis [33]. Our barriers were related to knowledge, skills and attitudes among the staff, the screening tool and lack of paper reminders [34–36]. A tailored intervention was planned to overcome the multiple identified barriers. This consisted of interactive-didactic workshops [16, 37, 38], the use of local opinion leaders [39–41], manual paper reminders [41] and feedback [42]. These multifaceted, tailored interventions are presented in Table 2. In addition we made some minor changes to the layout of the screening tool to make it easier to use [41].

**Table 2:** Implementation strategies

Interventions	Content of interventions
Workshop [16, 37, 38]	The learning activities consisted of an e-learning program, an interactive lecture, case studies and practical training in dysphagia screening. The content of the e-learning program was anatomy and physiology related to swallowing, and dysphagia, swallowing screening and measures aimed at swallowing deficits. In addition, we went through our local procedure on dysphagia screening. The workshop lasted for three days with an hour duration on the first 2 days, and 2 1/2 h on the last day. All nurses on the unit attended the workshop.
Local opinion leaders [39–41]	With the assistance of management, we identified local opinion leaders in the stroke unit. This was respected nurses with knowledge and skills in acute stroke treatment. The local opinion leaders were active throughout the entire implementation and re-audit period. They taught at the workshop, supervised novice nurses, administered the manual paper reminders and they talked about the importance of the project at the unit.
Manual paper reminders [41]	Checklists were used to remind the staff of swallow screening. We developed cards to put on the patient’s bedside table to remind the staff not to give the patient food or drink before swallow screening.
Feedback [42]	We gave feedback to the management and the staff on the level of care in plenaries. We did this one time on baseline and three times during data collection for re-audit. During these plenaries we discussed challenges and questions that related to the swallow screening.

### Ethical considerations

The project was approved by the Data Protection Authority in Helse Bergen Haukeland University Hospital according to The Health Personnel Act § 26 [43]. The authors declare that there are no competing interests.

### Statistical analysis

Our statistical calculations were performed using IBM SPSS Statistics (version 22.0). The significance level was set at 5%. We reported the demographic data as number and percentage, and as median, range and interquartile range. To assess homogeneity in patient characteristics, and to assess differences in swallow screening between baseline and re-audit, we used the Pearson Chi-Square test or exact test (if assumptions were not met) for categorical variables, and the Mann-Whitney *U* test for continuous variables. Observed differences were reported with 95% CI. We used the statistical software R version 2.15.0 (http://www.r-project.org/) to calculate 95% CI for the difference in proportions and median.

## Results

We analysed 88 EMRs at baseline and 51 in the re-audit. Two EMRs were excluded from the baseline because the patients were transferred to another hospital within few hours after admission. There was no statistically significant difference in median age (*p* = 0.1), gender (*p* = 0.3), ICD-10 diagnoses (*p* = 0.3) or stroke severity categories (*p* = 0.1) when comparing the patients at baseline to the re-audit. Median stroke severity was however somewhat higher for the baseline group (*p* = 0.007) (Table 3).

**Table 3:** Patients characteristics for baseline and the re-audit

	Baseline *n* = 88	Re-audit *n* = 51	*P* value*
Male, *n* (%)	51 (57)	24 (47)	0.293
Age			0.101
Median	79	83	
Interquartile range	66–88	76–87	
Range	39–96	23–100	
ICD-10 Code, *n* (%)			0.268
I61- Non-traumatic intracerebral hemorrhage	12 (14)	4 (8)	
I63-Cerebral infarction	49 (56)	25 (49)	
I64-Stroke, not specified as haemorrhage or infarction	0 (0)	0 (0)	
G45.9-Transient cerebral ischemic attack, unspecified (TIA)	27 (31)	22 (43)	
Stroke severity–NIHSS			
Median	2	1	0.007
Interquartile range	1–6	0–2	
Range	0–35	0–22	
Stroke severity–NIHSS, *n* (%)			0.140
Mild 0–8	62 (70)	44 (80)	
Moderate 9–16	8 (10)	1 (2)	
Severe > 18	8 (10)	3 (5)	

*Mann Whitney U test for continuous variables and Pearson Chi-Square test or exact test for categorical variables. Information on NIHSS was not available for 10 persons at baseline and seven persons at re-audit

I61-Non-traumatic intracerebral hemorrhage, I63-Cerebral infarction, I64-Stroke, not specified as haemorrhage or infarction and G45.9-Transient cerebral ischemic attack, unspecified (TIA).

The re-audit showed an improvement of 55%, (*p* < 0.001), in screening stroke patients for dysphagia compared to baseline (Table 4). Time from admission to swallow screening was documented in only 7% of the EMR at baseline but increased to 76% in re-audit (*p* < 0.001). The median time from admission to swallow screening was reduced by nearly four hours from baseline to re-audit (*p* = 0.02) (Table 4).

**Table 4:** Adherence to recommendation for swallow screening and documentation of time from admission to swallow screening

	Baseline *n* = 88	Re-audit *n* = 51	Difference (95% CI)	*P* value*
Adherence to recommendation				
Yes, *n* (%)	5 (6)	31 (61)	26 (55; 39–70)	<0.001
Time from admission to swallow screening				
Documented in EMR, *n* (%)	6 (7)	39 (76)	33 (69; 55–84)	<0.001
Median time, hour	6.1	2.3	3.8 (0.5–14.8)	0.024
Interquartile range	3.2–19.5	1.5–3.5		
Range	1.7–26.9	0.8–96.7		

*Mann Whitney U test for continuous variables and Pearson Chi-Square test for categorical variables

Documentation on whether patients were screened for swallow deficit before they received food or drink was found in 17 (31%) of the EMR at baseline and in 19 (45%) in re-audit (*p* = 0.24). Among the patients who were registered not screened, 59% had an incomplete screening at baseline and 50% at re-audit (*p* = 0.6).

## Discussion

This quality improvement project revealed a serious and large discrepancy in the care of acute stroke patients at baseline, when reviewed against evidence-based recommendations. Specifically, only 6% of patients admitted to the unit had their swallow ability examined. A systematic approach to develop a change strategy, tailored to local barriers and largely founded in implementation science, led to a significant improvement where this percentage increased to 61%. Other substantial improvements gained were the documentation of timing of screening, which increased from 7 to 76% and the time from admission to screening for dysphagia, which was reduced by nearly four hours.

Our project had several noteworthy strengths. First, we invested efforts in developing the audit tool, and later in testing it for validity. Also, we made sure that recommendations used to set the criteria, explicitly came from evidence based guidelines that we systematically searched for, which in turn were appraised for quality using the acknowledged AGREE II instrument. We believe that transparency in this process gave the project credibility among the staff in the unit, including the management. Later, we included them in the process of identifying the barriers and in the development of the multifaceted change strategy, in line with the success factors highlighted in the literature.

Our significant improvement is not unique if compared to other similar studies. In fact, most other studies aimed at improving screening for swallowing problems demonstrated a better care after intervening, with improvements between 35 and 58% [16, 17, 44]. Still, the post intervention result in our project showed a higher overall adherence than these other studies. This may be due to the difference in context in which we did our studies, such as in an emergency department [44], across a large hospital [17], or even in multi centres [16]. We anticipate that all of these settings are more complex to implement changes to practice, than ours. Unlike the aforementioned studies, our hospital is a small local hospital with only 90 stroke patients admitted annually. Our target group was the nurses conducting swallow screening, and the swallow screening tool had already been introduced prior to our first audit. Where the healthcare professionals targeted in referred studies were dealing with so much more than stroke patients on a daily basis, our target group consisted of nurses assessing stroke patients for swallowing problem. This may explain partly why sustainability was poor in several of the studies [16, 44]. Also adding complexity may be if the goal is not only to improve screening for dysphagia, but extends to other areas of stroke care too [16]. When Hinchey et al. discussed reasons for only gaining smaller amounts of improvement than hoped for, or the lack of sustainability, then reasons like lack of involvement of physicians or leaders, a short intervention time frame, and a lack of resources for the implementation were listed [45].

Quality improvement projects must always be assessed cautiously for generalizability, due to its local contextual factors. Still, we believe that many of our success factors may be of importance and relevance in other settings and projects. The overarching factor of success in our project was to contextualize the project by targeting local barriers and facilitators and to adapt implementation strategies from implementation science that fit the barriers profile of the setting. In this work, we involved the health care professionals and their leaders for who we expected to change their behaviour. There are some threats to the internal validity of our project. The most important one is the chance of a Hawthorne effect, where the health care professionals in our unit may have adjusted their behaviour as they knew that they were being observed for a certain time period. We believe that the risk of bias in the baseline and the re-audit measurements was small. This is due to the same trained person collecting data [28], the data was verified twice and because there was an instruction to the audit tool [31].

Since we did not have control over other factors than our intervention, a causative association between intervention and result cannot be used to draw definitive conclusions [28]. However, we are not aware of any other intervention or factors, besides those we implemented, which could have affected the outcome of this CBCA. It is therefore likely that our implementation strategy led to this increase in adherence.

## Conclusions

This criteria-based clinical audit proved successful in improving adherence to evidence based recommendations on swallow screening in the care for acute stroke patients. Improving processes of care to identify dysphagia in the acute phase of a stroke, is the first and necessary step to reduce fatal consequences of aspiration pneumonia. We believe that our tailored implementation strategy, developed to fit the local context, has practical relevance for improving the quality and safety of stroke care in other contexts.

## Abbreviations

CBCA: Criteria-based clinical audit
CI: Confidence interval
CVI: Content validity index
I-CVI: Content validity of individual items
S-CVI: Content validity, average of all items of the too
EMR: Electronic medical records
NIHSS: The National Institutes of Health Stroke Scale
